# Supplementary material for: Repurposing FDA Drug Compounds against Breast Cancer by Targeting EGFR/HER2
Source: Pharmaceuticals (Basel). 2021 Aug 12;14(8):791. doi: 10.3390/ph14080791 (PMC8401258; doi:10.3390/ph14080791)
Supplement: Supplementary file 1 [file pharmaceuticals-14-00791-s001.zip › pharmaceuticals-1324384-supplementary.pdf]

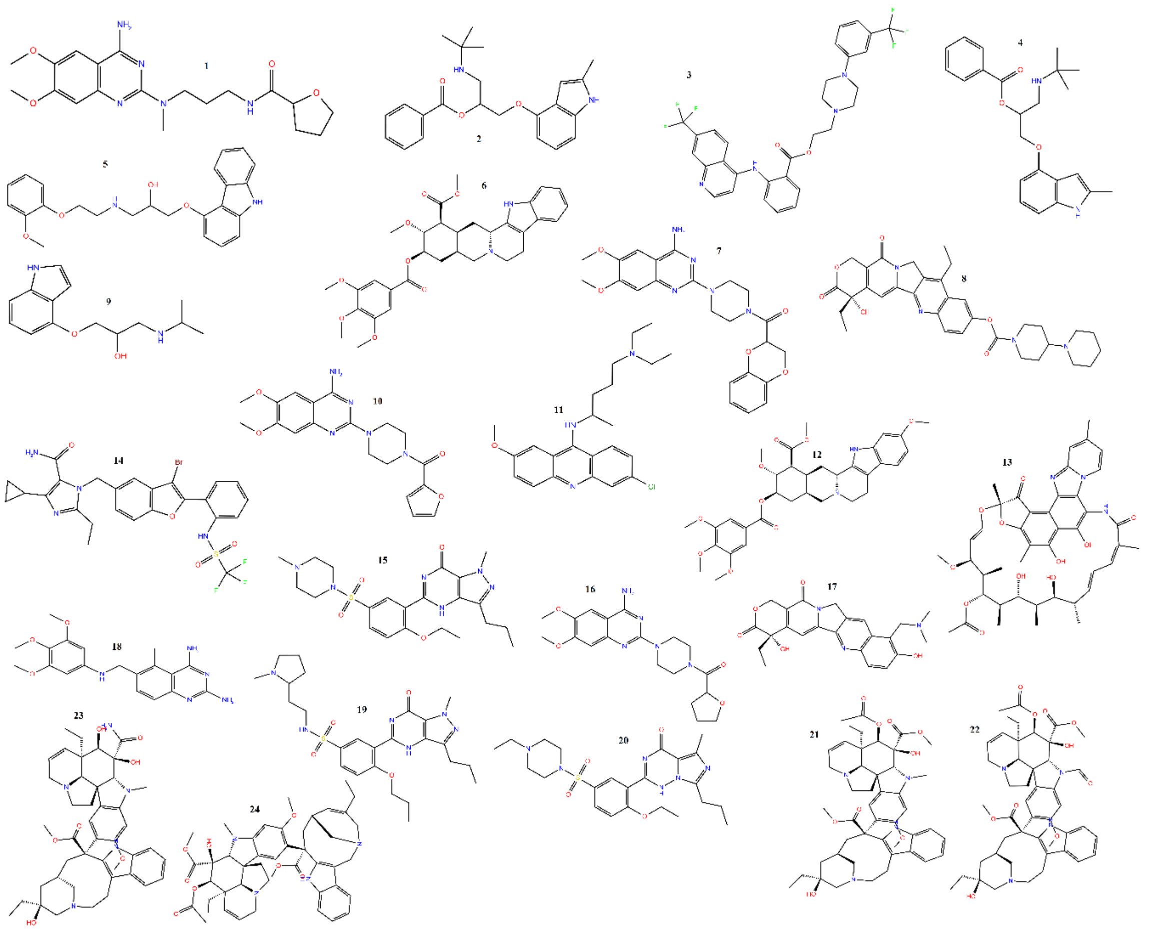

Scheme S1. FDA-drugs with structural similarity with lapatinib and gefitinib. Alfuzosin (1), amodiaquine (2), antrafenine (3), bopindolol (4), carvedilol (5), deserpidine (6), doxazosin (7), irinotecan (8), pindolol (9), prazosin (10), quinacrine (11), rifaximin (12), sildenafil (13), reserpine (14), saprisartan (15), terazosin (16), topotecan (17), trimetrexate (18), udenafil (19), vardenafil (20), vinblastine (21), vincristine (22), vindesine (23), and vinorelbine (24).

Table S1. Docking studies of candidates to inactive EGFR/HER2 inhibitors selected in the DrugBank.

Drug	Docking score for HER2	Docking score for EGFR
Alfuzosin	-9.545385	-9.152678
Amodiaquine	-8.069709	-7.6083474
Antrafenine	-9.403273	-7.9908357
Bopindolol	-7.478974	-8.168499
Carvedilol	-8.012247	-7.6812377
Deserpidine	ND	-8.787144
Doxasozin	-7.6163125	-7.927016
Irinotecan	-8.696633	-8.757377
Pindolol	-6.8409348	-6.9343553
Prazosin	-8.887536	-8.209263
Quinacrine	-8.366776	-8.351075
Reserpine	-9.471398	-9.074252
Rifaximin	ND	ND
Saprisartan	-9.437706	-8.517011
Sildenafil	-8.113859	-8.675298
Terazosin	-9.387002	-8.282405
Topotecan	-7.7922587	-7.7152147
Trimetrexate	-9.071067	-7.3534374
Udenafil	-9.14161	-7.403184
Vardenafil	ND	-8.327767
Vinblastine	ND	ND
Vincristine	ND	ND
Vindesine	ND	ND
Vinorelbine	ND	ND

ND: No docking was observed.

Table S2. Interacting residues between inhibitors selected in the DrugBank with HER2.

Drug	Docking score for HER2
Alfuzosin	L726, F731, V734, A751, I752, K753, M774, S783, R784, L785, L796, V797, T798, Q799, L800, M801, Y803, G804, C805, L852, T862, D863, F864.
Amodiaquine	L726, G727, F731, V734, A751, K753, T798, Q799, L800, M801, P802, Y803, G804, C805, R849, N850, L852, T862, D863.
Antrafenine	L726, F731, V734, A751, I752, K753, M774, S783, L785, L796, V797, T798, L800, M801, Y803, G804, C805, D808, H809, L852, V853, T862, D863, F864.
Bopindolol	L726, G727, F731, V734, A751, K753, S783, T798, Q799, L800, M801, P802, Y803, G804, C805, L852, T862, D863.
Carvedilol	L726, F731, V734, A751, K753, M774, S783, L785, L796, T798, Q799, L800, M801, Y803, G804, C805, L852, T862, D863, F864.
Doxasozin	V725, L726, S728, G727, K736, V734, K753, G804, C805, L807, D808, R849, N850, L852, T862, D863, L866
Irinotecan	L726, F731, V734, A751, K753, V754, L755, R756, I767, M774, S783, L785, L796, V797, T798, Q799, L800, M801, P802, Y803, G804, C805, L852, T862, D863, F864, G865.
Pindolol	L726, V734, A751, I752, K753, M774, S783, L785, L796, V797, T798, Q799, L800, M801, Y803, G804, L852, T862, D863, F864.
Prazosin	L726, F731, V734, A751, I752, K753, R756, M774, S783, R784, L785, L796, V797, T798, M801, Y803, G804, C805, L852, T862, D863, F864.
Quinacrine	L726, F731, V734, A751, I752, K753, M774, S783, L785, L796, V797, T798, Q799, L800, M801, Y803, G804, C805, R849, N850, L852, T862, D863, F864.
Reserpine	L726, F731, V734, A751, K753, V754, M774, S783, R784, L785, L796, T798, Q799, L800, M801, Y803, G804, C805, D808, H809, R849, N850, V851, L852, T862, D863, F864.
Saprisartan	L726, F731, V734, A751, I752, K753, V754, L755, R756, M774, S783, L785, L796, V797, T798, Q799, L800, M801, P802, Y803, G804, C805, L852, T862, D863, F864.
Sildenafil	L726, F731, V734, A751, K753, S783, L785, L796, V797, T798, Q799, L800, M801, Y803, G804, C805, L852, T862, D863, F864.
Terazosin	L726, F731, V734, A751, I752, K753, M774, S783, R784, L785, L796, V797, T798, Q799, L800, M801, Y803, G804, C805, L852, T862, D863, F864.
Topotecan	L726, F731, V734, A751, K753, S783, T798, Q799, L800, M801, Y803, G804, C805, D808, L852, T862, D863.
Trimetrexate	F731, V734, A751, I752, K753, M774, S783, R784, L785, L796, V797, T798, M801, Y803, G804, C805, L852, T862, D863, F864.
Udenafil	L726, G727, F731, V734, A751, I752, K753, S783, L785, L796, V797, T798, L800, M801, P802, Y803, G804, C805, D808, L852, T862, D863.

Table S3. Interacting residues between inhibitors selected in the DrugBank with EGFR.

Drug	Docking score for EGFR
Alfuzosin	L718, V726, K728, A743, K745, M766, C775, R776, L777, L788, T790, Q791, L792, M793, P794, G796, C797, D800, R841, L844, N842, T854, D855, F856, L858.
Amodiaquine	L718, G719, V726, K728, A743, Q791, L792, M793, P794, F795, G796, C797, L799, D800, R803, R841, L844.
Antrafenine	L718, G719, S720, V726, A743, K745, T790, Q791, L792, M793, G796, C797, R841, L844, N842, T854, D855.
Bopindolol	L718, V726, A743, I744, K745, L777, L788, I789, T790, Q791, L792, M793, G796, C797, D800, R841, L844, N842, T854, D855, F856, L858.
Carvedilol	L718, G719, S720, G721, A722, F723, G724, T725, V726, A743, K745, D746, L747, Q791, L792, M793, P794, G796, C797, R841, L844, G873, K875.
Deserpidine	L718, S720, G721, A722, F723, G724, T725, V726, A743, K745, E746, L747, L792, M793, P794, F795, G796, C797, D800, R841, L844, T854, G873, G874, K875.
Doxasozin	L718, G719, S720, V726, A743, K745, C775, T790, Q791, L792, M793, G796, C797, D800, L844, T854, D855.
Irinotecan	L718, G719, S720, G721, A722, F723, G724, T725, V726, K728, K745, L747, L792, M793, P794, F795, G796, G873, G874, K875.
Pindolol	L718, V726, A743, K745, T790, Q791, L792, M793, G796, C797, R841, N842, L844, T854, D855.
Prazosin	L718, V726, A743, I744, K745, M766, C775, L777, L788, T790, L792, M793, P794, F795, G796, C797, D800, R841, L844, T854, D855, F856, L858.
Quinacrine	L718, V726, A743, K745, T790, Q791, L792, M793, P794, G796, C797, D800, R841, L844, T854, D855, L858.
Reserpine	L718, G719, G721, A722, F723, V726, K728, A743, K745, L792, M793, P794, G796, C797, D800, R841, N842, L844, T854, G873, G874, K875.
Saprisartan	L718, G719, V726, A743, K745, T790, Q791, L792, M793, G796, C797, L799, R803, R841, L844, T854, D855, K875.
Sildenafil	L718, G719, S720, G721, A722, F723, G724, T725, V726, K745, G796, C797, L799, D800, R841, G873, G874, K875.
Terazosin	L718, V726, K728, A743, I744, K745, M766, L777, L788, T790, L792, M793, P794, G796, C797, D800, R841, L844, T854, D855, F856, L858.
Topotecan	L718, V726, K728, A743, K745, C775, T790, L792, M793, P794, G796, L844, T854, D855.
Trimetrexate	L718, G719, S720, G721, A722, V726, A743, K745, L792, M793, G796, C797, R841, L844, T854, D855, G873, K875.
Udenafil	L718, G719, S720, G721, V726, K728, K745, T790, Q791, L792, M793, P794, G796, C797, R841, L743, L844, T854, D855, G873, K875.
Vardenafil	L718, G719, S720, G721, A722, F723, G724, T725, V726, A743, K745, C775, T790, L792, M793, G796, C797, D800, R841, L844, T854, D855.

Table S4. Average RMSD and RG values (Å) during the last 50 ns of MD simulations.

System	RMSD	RG
$HER2_{alfuzosin}$	1.81 ± 0.15	19.87 ± 0.08
$HER2_{antrafenine}$	1.94 ± 0.37	19.92 ± 0.08
$HER2_{bopindolol}$	2.15 ± 0.19	19.88 ± 0.09
$HER2_{carvedilol}$	2.61 ± 0.30	19.91 ± 0.09
$HER2_{doxasozin}$	3.47 ± 0.40	20.24 ± 0.11
$HER2_{irinotecan}$	2.39 ± 0.20	20.10 ± 0.10
$HER2_{pindolol}$	2.24 ± 0.31	19.80 ± 0.08
$HER2_{prazosin}$	2.15 ± 0.16	19.93 ± 0.07
$HER2_{quinacrine}$	2.01 ± 0.28	19.92 ± 0.07
$HER2_{saprisartan}$	2.98 ± 0.20	20.02 ± 0.07
$HER2_{sildenafil}$	2.34 ± 0.12	20.01 ± 0.06
$HER2_{terazosin}$	3.19 ± 0.33	20.04 ± 0.11
$HER2_{topotecan}$	2.17 ± 0.15	19.73 ± 0.07
$HER2_{trimetrexate}$	2.34 ± 0.33	19.83 ± 0.08
$EGFR_{alfuzosin}$	1.94 ± 0.11	18.90 ± 0.06
$EGFR_{amodiaquine}$	2.05 ± 0.19	19.06 ± 0.11
$EGFR_{antrafenine}$	1.84 ± 0.15	19.00 ± 0.08
$EGFR_{bopindolol}$	2.04 ± 0.14	19.06 ± 0.08
$EGFR_{carvedilol}$	2.06 ± 0.28	18.97 ± 0.08
$EGFR_{doxasozin}$	2.39 ± 0.17	19.06 ± 0.12
$EGFR_{pindolol}$	1.97 ± 0.11	19.19 ± 0.07
$EGFR_{prazosin}$	2.09 ± 0.08	19.17 ± 0.07
$EGFR_{quinacrine}$	1.93 ± 0.26	19.12 ± 0.08
$EGFR_{saprisartan}$	1.65 ± 0.15	18.98 ± 0.07
$EGFR_{terazosin}$	1.78 ± 0.11	19.00 ± 0.09
$EGFR_{topotecan}$	1.96 ± 0.22	19.11 ± 0.09
$EGFR_{trimetrexate}$	2.19 ± 0.20	19.24 ± 0.09
$EGFR_{udenafil}$	2.11 ± 0.11	18.91 ± 0.07
$EGFR_{vardenafil}$	2.03 ± 0.18	18.93 ± 0.13

Table S5. FDA-drugs with structural similarity with lapatinib and gefitinib.

Drug	DrugBank Accession Number
Alfuzosin	DB00346
Amodiaquine	DB00613
Antrafenine	DB01419
Bopindolol	DB08807
Carvedilol	DB01136
Deserpidine	DB01089
Doxasozin	DB00590
Irinotecan	DB00762
Pindolol	DB00960
Prazosin	DB00457
Quinacrine	DB01103
Reserpine	DB00206
Rifaximin	DB01220
Saprisartan	DB01347
Sildenafil	DB00203
Terazosin	DB01162
Topotecan	DB01030
Trimetrexate	DB01157
Udenafil	DB06267
Vardenafil	DB00862
Vinblastine	DB00570
Vincristine	DB00541
Vindesine	DB00309
Vinorelbine	DB00361
